# 

**Published:** 1852-04

**Authors:** 


### RECEIPTS FOR THE REGISTER.

We give below a list of those subscribers who have paid for Vol. V. of the Register, and shall continue this in each number, so that all will know how their account stands.

| | |
|---|---|
| Dr. J. A. Weaver, Batavia, - - - | $2 00 |
| " Sam. Griffith, Louisville. Ky., - - - | 2 00 |
| " W. C. Duncan, Cincinnati, O., - - - | 2 00 |
| " D. Dougherty, Bardstown, Ky., - - - | 2 00 |
| " C. T. Cushman, Columbus, Ga., - - - | 2 00 |
| " J. P. Ulrey, Rising Sun, Ia., - - - | 2 00 |
| " H. R. Smith, Terre Haute, Ia., - - - | 2 00 |
| " J. Scott, Pittsburg, Pa., - - - - | 2 00 |
| " G. W. Keely, Oxford, O., - - - | 2 00 |
| " G. Y. Shirley, Jacksonville, Ill., - - - | 2 00 |
| " J. S. Curtis, Vincennes, Ia., - - - | 2 00 |
| " B. Wood, Nashville, Tenn., - - - | 2 00 |
| " T. F. Compton, Hillsboro, O., - - - | 2 00 |
| " E. C. Buckwell, Zanesville, O., - - - | 2 00 |
| " E. S. Holmes, Lockport, N. Y., - - - | 2 00 |
| " A. J. Reeves, Mt. Vernon, O., - - - | 2 00 |
| " James S. Knapp, New Orleans, La., - - | 2 00 |
| " W. Perrin, Springfield, O., - - - | 2 00 |
| " J. B. Dunlevy, Pittsburg, Pa., - - - | 2 00 |
| " N. W. Hicks, Keokuk, Iowa, - - - | 2 00 |
| " J. M. Abrams, Brownsville, Pa., - - - | 2 00 |
| " J. F. Wilson, Greencastle, Ia., - - - | 2 00 |
| " N. P. Allen, Smith Grove, Ky., - - - | 2 00 |
| " W. A. Pease, Dayton, O., - - - | 2 00 |
| " J. Waylan, Lancaster, Pa., - - - | 2 00 |
| " A. F. Ennis, S. Joseph, Mo., - - - | 2 00 |
| Mr. Post, Cincinnati, O., - - - - | 2 00 |
| Dr. J. C. Whinery, Salem, O., - - - | 2 00 |
| " John H. Stark, Independence, Mo., - - | 2 00 |
| " E. Stone, Houston, Texas, - - - | 2 00 |
| " J. H. Olds, Circlevile, "O - - - | 2 00 |
| " Jacob McErven, Noble's Town, Pa., - - - | 2 00 |
| " R. D. Booth, Middletown, O., - - | 2 00 |
| " H. W. Robbins, Ludlow, Miss., - - - | 2 00 |
| " H. B. Sisson, Patriot, Ia., - - - | 2 00 |
| " J. Hardman, Salineville, O., - - - | 2 00 |

EIGHTH ANNUAL ANNOUNCEMENT OF

### THE OHIO COLLEGE OF DENTAL SURGERY.

### SESSION OF 1852--'53.

The regular course of Lectures in this Institution commences the first Monday of November and closes on the 20th of February.

FACULTY:

**JAMES TAYLOR, M.D., D.D.S.,**

*Professor of the Principles and Practice of Dental Surgery.*

**THOMAS WOOD, M.D.,**
*Professor of Anatomy amd Physiology.*

**GEORGE MENDENHALL, M.D.,**
*Professor of Pathology and Therapeutics.*

**JOHN ALLEN, D.D.S.**
*Professor of Operative and Mechanical Dentistry.*

**G. L. VAN EMON, A.M., D.D.S.,**
*Lecturer on Dental Chemistry and Demonstrator of Operative and Mechanical Dentistry.*

EXAMINING COMMITTEE.—DENTAL.

**Dr. R. Somerby,** Louisville, Ky.,

" **E. Hale,** St. Louis, Mo.,

" **A. L. Talbert,** Dayton, Ohio.

MEDICAL.

**Dr. P. S. Conner,** Cincinnati, Ohio,
" **P. G. Fore,** " "

The Faculty, in issuing this their eighth annual announcement, take pleasure in stating that the Institution is established on a permanent basis, and is in a flourishing condition. They feel that the difficulties which present themselves in the establishment of a new Institution of the kind, have been overcome, and that now a systematic course of instruction has been obtained which shall meet the wants and necessities of the profession.

A building, suitable in every respect for the purposes of a Dental College, is now owned by the profession and dedicated to the advancement of Dental Science.

A fund has been appropriated for the purchase of a Library, and such apparatus as may from time to time appear necessary.

The arrangements in the Laboratory are such as to afford every facility in Mechanical Dentistry, and a demonstrator of experience and ability takes charge of this department.

| | |
|---|---|
| Tickets for the entire course, - - - - - | $100 00 |
| Matriculating Ticket, - - - - - - - - | 5 00 |
| Diploma fee, - - - - - - - - - - - | 25 00 |

Good board can be had from $2 to $3 per week.

JAMES TAYLOR, *Dean.*

